# Coordinated Expression of Ras Suppressor 1 (RSU-1) and Growth Differentiation Factor 15 (GDF15) Affects Glioma Cell Invasion

**DOI:** 10.3390/cancers11081159

**Published:** 2019-08-13

**Authors:** Maria Louca, Vasiliki Gkretsi, Triantafyllos Stylianopoulos

**Affiliations:** 1 Cancer Biophysics Laboratory, Department of Mechanical and Manufacturing Engineering, University of Cyprus, 1678 Nicosia, Cyprus; 2 Biomedical Sciences Program, Department of Life Sciences, School of Sciences, European University Cyprus, 1516 Nicosia, Cyprus

**Keywords:** Ras Suppressor 1 (RSU-1), growth differentiation factor 15 (GDF15), cell-extracellular matrix adhesion, PINCH1, RhoA, actin cytoskeleton, migration, invasion

## Abstract

Glioblastoma multiforme (GBM) is the most aggressive type of brain tumor due to its invasive phenotype. Ras suppressor 1 (RSU-1) is a cell-extracellular matrix adhesion protein and we recently found that it promotes cell invasion in aggressive cells and inhibits it in non-invasive. Growth differentiation factor-15 (GDF15) is known to be involved in actin cytoskeleton reorganization and metastasis. In this study, we used three brain cell lines (H4, SW1088 and A172) with increasing *RSU-1* expression levels and invasive capacity and decreasing *GDF15* levels to investigate the interplay between RSU-1 and GDF15 with regard to cell invasion. Four experimental approaches were used: (a) GDF15 treatment, (b) *Rsu-1* silencing, (c) *GDF15* silencing, and (d) combined GDF15 treatment and *RSU-1* silencing. We found that the differential expression of *RSU-1* and *GDF15* in H4 and A172 cells leading to inhibition of cell invasion in H4 cells and promotion in A172 through respective changes in *PINCH1*, *RhoA* and *MMP-13* expression. Interestingly SW1088, with intermediate *RSU-1* and *GDF15* expression, were not affected by any treatment. We conclude that there is a strong connection between RSU-1 and GDF15 in H4, SW1088 and A172 cells and the relative expression of these two proteins is fundamental in affecting their invasive fate.

## 1. Introduction

Glioblastoma multiforme (GBM) is the most aggressive type of brain tumor, which is known to develop from astrocytes and to be associated with poor prognosis and decreased survival rates [1,2]. It is characterized by the ability of the cells to invade the surrounding brain parenchyma [3,4,5]. Its aggressive nature makes the identification of the precise molecular mechanism involved in its pathogenesis imperative. A number of studies have given emphasis to the significance of cell-extracellular matrix (ECM) interactions on the progression and invasive potential of human astrocytic tumors [6,7,8]. More specifically, focal adhesion (FA) proteins, localized at cell-ECM adhesion sites maintain direct or indirect connections with actin cytoskeleton [9,10] and thus, they are critically involved in many physiological and pathological processes including the regulation of the migratory and invasive capacity of glioma cells [11,12]. Therefore, alterations in the expression or function of FA proteins directly affects the ability of glioma cells to migrate and invade into adjacent tissues of the brain.

Ras suppressor-1 (RSU-1) is a FA protein initially identified as suppressor of Ras-dependent oncogenic transformation [13,14,15,16,17] but it was recently shown to interact with the LIM5 domain of the Particularly interesting new cysteine-histidine rich protein (PINCH-1) at FA sites [18,19]. PINCH1 directly binds to Integrin-linked kinase (ILK), which in turn binds to alpha-parvin (PARVA) forming a stable ternary complex at FAs that is also tightly connected to the actin cytoskeleton through direct interaction of PARVA with actin [20,21,22]. Apart from the effect of FAs on actin cytoskeleton reorganization, Rho-GTPases which are downstream targets of Ras, are also involved in actin cytoskeleton reorganization and have been implicated in glioma cell migration, and invasion, as well as in tumor progression [23,24,25]. Also, several recent studies have indicated that *RSU-1* silencing inhibits migration and invasion of hepatocellular carcinoma, breast and colon cancer cells [26,27,28,29,30]. Despite the fact that there is a connection between *Ras* oncogene and cancer cell aggressiveness, the exact role of RSU-1 with regard to the metastatic properties of cancer cells remains unclear.

Regarding the role of RSU-1 in the central nervous system [31,32,33], not much is known either. Interestingly though, our recent work demonstrated a differential regulation of cell migration and invasion of glioma cells by RSU-1 based on their aggressiveness [34]. Thus, RSU-1 was shown to promote the invasion capacity of aggressive glioma cells (A172 and U87-MG) but inhibit that of non-aggressive cells (H4 and SW1088), indicating that a complex molecular mechanism is in place. 

Growth differentiation factor (GDF15), also known as macrophages inhibitory cytokine (MIC-1) [35], Placental bone morphogenetic protein (PLAB) [36], Placental transforming growth factor B (P-TGFβ) [37], Prostate-derived factor (PDF) [38], and Non-steroidal anti-inflammatory drug-activated gene-1 (*NAG1*) [39] is a member of the Transforming growth factor beta (TGF-β) superfamily of proteins known to be secreted in low levels in all normal tissues other than placenta in which it is quite abundant [40]. Interestingly, GDF15 has been reported to be involved in actin cytoskeleton reorganization and remodeling [41] while at the same time being implicated in the regulation of proliferation and invasion in breast, prostate, colon, liver and pancreatic cancer cells [42,43,44,45,46,47,48]. More importantly, glioblastoma patients have been shown to have increased GDF15 levels in their blood [48] while higher *GDF15* mRNA expression inside the tumor has been associated with poor survival [49], suggesting that GDF15 likely possesses tumor-promoting properties. On the other hand, there has been evidence that GDF15 acts as tumor suppressor in glioma cells [50,51]. 

Taking all the above into consideration, the role of GDF15 with regard to cancer cell development and progression is still vague and it could depend on the cell-type, its expression levels or its interaction with other proteins [52,53]. 

In a recent in vitro study performed in breast cancer cells, we showed that *RSU-1* silencing downregulates several actin-modulating genes, namely *PARVA*, *RhoA*, *Rho associated kinase-1 (ROCK)* and *Fascin-1* and leads to inhibition of breast cancer cell migration and invasion [54]. Notably, however, treatment with human recombinant GDF15 (hrGDF15) completely reverses both the inhibition in gene expression and the functional effects on cell migration and invasion [54].

As this connection, between RSU-1 and GDF15 is not yet well-defined, in the present study, we investigated the interplay between RSU-1 and GDF15 in glioma cell lines and the effect of their expression on glioma cell migration and invasion.

## 2. Results

### 2.1. Growth Differentiation Factor 15 (GDF15) mRNA Expression is Reduced in More Aggressive Glioma Cells

**Figure 1:**
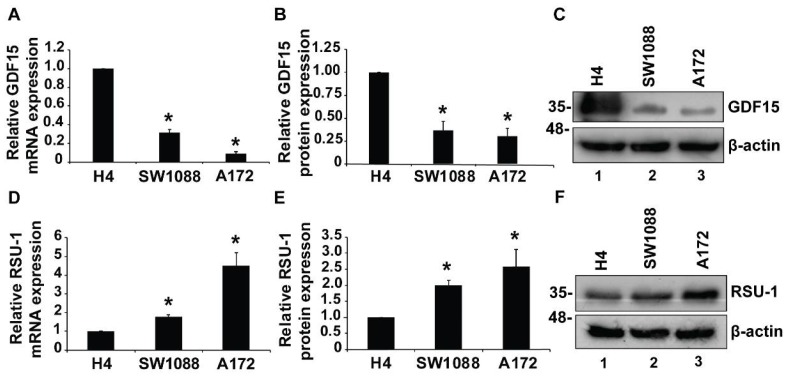
Growth differentiation factor (*GDF15*) expression decreases from the less aggressive (H4) towards the more aggressive (A172) cells, whereas the RSU-1 expression follows the opposite pattern. (**A**–**D**) Relative *GDF15* and *RSU-1* mRNA expression in three brain cell lines (H4, SW1088 and A172). Three independent real-time polymerase chain reaction (PCR) experiments were performed. (**B**–**E**) Western blot for GDF15 and RSU-1 protein expression with H4 cell line as the sample control and β-actin as the loading control. (**C**–**F**) Graphs show the quantification of GDF15 and RSU-1 protein expression with ImageJ software from two different Western blots. Asterisks denote a statistically significant difference (*p* < 0.05) compared to the H4 data.

Since the role of GDF15 in cancer progression is controversial and not fully elucidated yet [50,51], we first tested *GDF15* expression in three cell lines H4, SW1088, and A172 both at the mRNA (Figure 1A) and protein level (Figure 1B,C). In our previous work [34], we have shown that A172 cells that cause GBM are very aggressive having a strong invasive capacity in contrast to SW1088 cells, which cause astrocytoma and are less invasive, and H4 cells which are almost non-invasive neuroglioma cells. Here, we show that H4 cells express *GDF15* at higher levels than SW1088 and A172 cells both at the mRNA and protein level (Figure 1), whereas *RSU-1* expression follows the exact opposite pattern, being elevated as the aggressiveness of cells increases (Figure 1D–F).

Intrigued by this finding, we wondered whether *RSU-1* and *GDF-15* are collaborating in regulating glioma cell invasion through a common molecular pathway, as both genes are indirectly associated with actin cytoskeleton reorganization and aggressive cancer cell behavior [21,45].

### 2.2. Human Recombinant GDF15 (hrGDF15) Treatment Protein Differentially Affects Motility and Invasive Capacity of Cells Depending on Cell Aggressiveness

**Figure 2:**
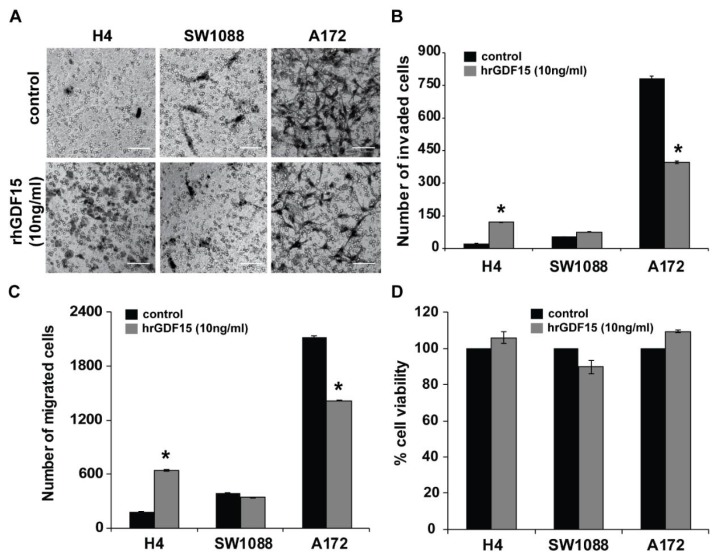
hrGDF15 treatment promotes migration and invasion of less invasive cells and inhibits that of the highly invasive cells without affecting cell survival. (**A**) Control and hrGDF15 (10 ng/mL) treated cells (H4, SW1088 and A172) were subjected to transwell invasion assay 24 h post-treatment. Scale bar: 100 μm. (**B**) Diagrammatic representation of results from invasion assays which depicts the total number of invading glioma cells per transwell for each group (nine randomly chosen microscopic fields per transwell). (**C**) Diagram showing the total number of migrated cells per transwell. Supplementary Figure S1 shows representative images of migration through the transwell for H4, SW1088 and A172 cells. For the invasion and migration assays three independent experiments were performed and each sample was run in duplicate. (**D**) Graph representing the percentage of cell viability as measured by Alamar blue assay 24 h post hrGDF15 treatment for the three cell lines. Each sample was run in triplicate and three independent experiments were performed. Asterisks denote a statistically significant difference (*p* < 0.05).

To evaluate the role of GDF15 in regulating the motility and invasion of glioma cells, H4, SW1088, and A172 cells were treated with human recombinant hrGDF15 (10 ng/mL) for 24 h and were then subjected to transwell migration and invasion assays. As shown in Figure 2, hrGDF-15 treatment increased both the invasion (Figure 2A,B) and migration (Figure 2C and Supplementary Figure S1) of the less invasive H4 cells and inhibited that of the more invasive A172 cells without any alteration in cell viability (Figure 2D). Notably, SW1088 cells, which have intermediate invasive capacity and *GDF15* basal level expression, did not show any statistically significant changes in migration (Figure 2C and Supplementary Figure S1) and invasion (Figure 2A,B) following hrGDF15 treatment. 

**Figure 3:**
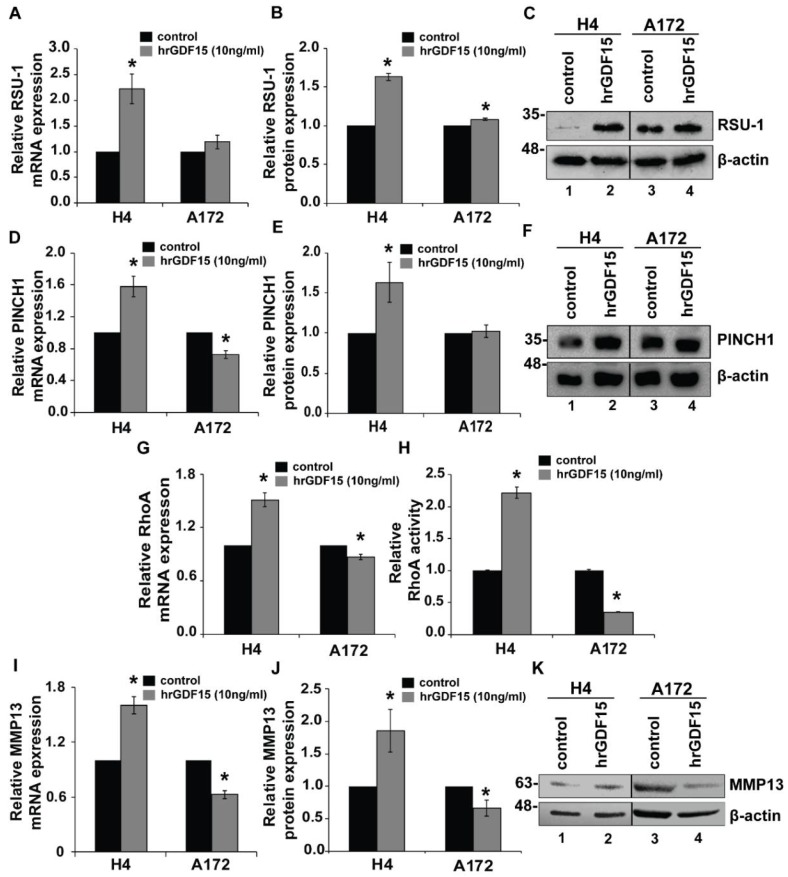
hrGDF-15 treatment upregulates *RSU-1* expression and promotes or suppresses invasion of glioma cells through upregulation or downregulation of PINCH1, RhoA, and MMP13 respectively. (**A**,**D**,**G**,**I**) Relative mRNA expression of *RSU-1*, *PINCH1*, *RhoA* and *MMP13* respectively in H4 and A172 cell line upon treatment with hrGDF15 (10 ng/mL) for 24 h. Four independent real-time PCR experiments were performed and data were analyzed using the ΔΔCt method using control-treated cells as a calibrator sample for each cell line. (**H**) Relative RhoA activity 24 h post rhGDF15 treatment on H4 and A172 cell lines. (**B**,**E**,**K**) Representative pictures of Western blot displaying RSU-1, PINCH1 and MMP13 protein expression following hrGDF15 treatment for 24 h. (**C**,**F**,**J**) Graphs representing quantification of RSU-1 and PINCH1 protein expression respectively for each cell line using ImageJ software and β-actin as loading control. Mean band intensity from two immunoblots from independent experiments was used for the quantification. Asterisks indicate a statistically significant difference (*p* < 0.05) compared to control data.

In order to identify possible connections between GDF15 and RSU-1 with regard to glioma cell invasion, we examined the *RSU-1* mRNA expression upon hrGDF-15 treatment and found that in H4 cells, which express low *RSU-1* and high *GDF-15* levels, *RSU-1* was strongly upregulated following hrGDF-15 treatment both at the mRNA (Figure 3A) and protein level (Figure 3B,C). By contrast, in A172 cells that already express high *RSU-1* and low *GDF15* levels, hrGDF15 treatment affected *RSU-1* expression to a lesser extent, presumably due to the fact that it is already highly expressed in these cells. 

Since RSU-1 is known to directly interact with PINCH1 [18,19], we wondered whether *PINCH1* expression is also affected by hrGDF15 treatment. As shown in Figure 3, hrGDF15 treatment led to upregulation of *PINCH1* in H4 cells which was not true for A172 cells (Figure 3D–F).

Subsequently, to determine whether the increased invasiveness of H4 cells and the decreased invasiveness of A172 cells observed upon hrGDF15 treatment involved Rho GTPases activity, we assessed RhoA activity by a G-LISA RhoA activation assay, as RhoA is known to be modulated by *Ras* oncogene and plays central role in actin cytoskeleton reorganization [55]. RhoA activity was increased in H4 cells and decreased in A172 cells compared to the control (Figure 3H), following an identical expression pattern (Figure 3G) with that of *PINCH1* (Figure 3D). We also tested whether major proteases are also affected by hrGDF15 treatment, and thus we examined the expression of *Matrix metalloproteinase 13 (MMP13)*, a metalloproteinase with a crucial role in glioma cell invasion as well as in other cancer types. Consistent with the changes observed in cell invasion, we found that *MMP13* mRNA (Figure 3I) and MMP13 protein (Figure 3K) in H4 and A172 cell lines follows the exact same pattern. Supplementary Figure S2 shows the relative mRNA expression for *RSU-1*, *PINCH1*, *MMP13* and *RhoA* in SW1088 cells, which express intermediate levels of both RSU-1 and GDF15, following hrGDF15 treatment. No clear inhibitory or promoting pattern can be observed in any of the genes tested in SW1088 which supports the invasion and migration results showing no effect of hrGDF15 treatment on these cellular properties (Figure 2).

### 2.3. RSU-1 Silencing Regulates GDF15 Expression and Differentially Affectscell Invasiveness and the Expression of PINCH1, RhoA and MMP13

**Figure 4:**
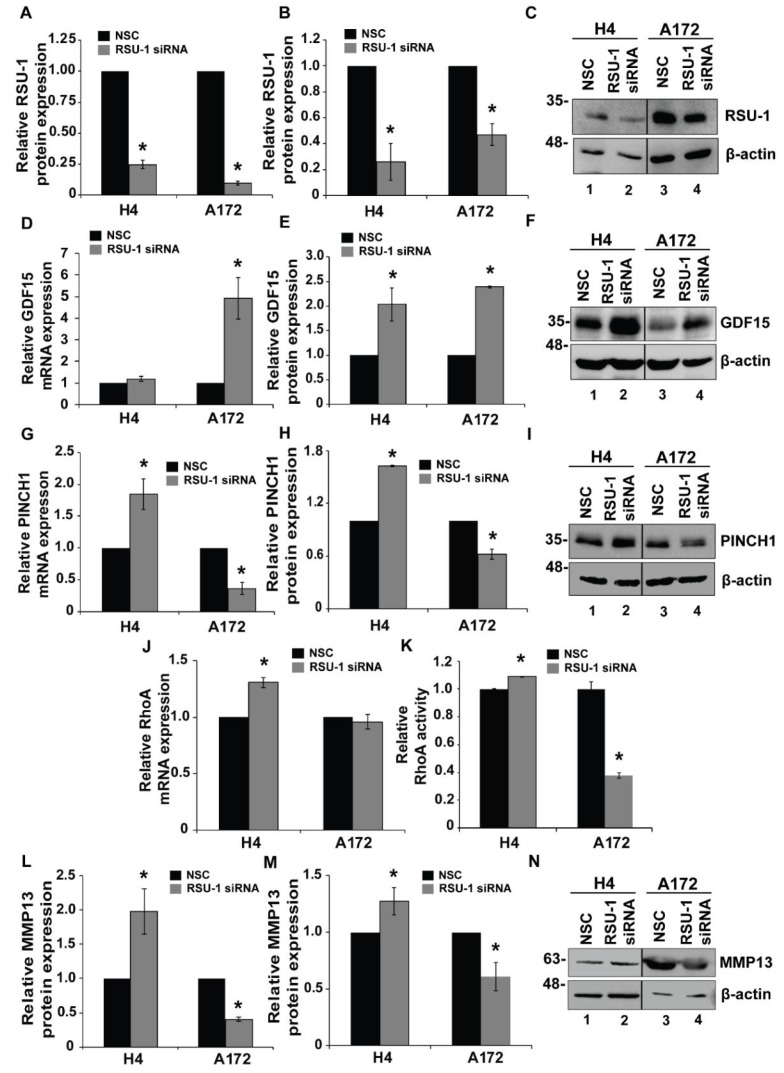
*RSU-1* silencing upregulates *GDF15* expression and regulates *PINCH1*, *RhoA* and *MMP13* expression. (**A**,**D**,**G**,**J**,**L**) Relative mRNA expression of *RSU-1*, *GDF15*, *PINCH1*, *RhoA* and *MMP13* respectively in the H4 and A172 cell lines upon *RSU-1* silencing. Four independent real-time PCR experiments were performed and data were analyzed using the ΔΔCt method, while non-specific control (NSC) treated cells were used as the calibrator sample for each cell line. (**B**,**E**,**H**,**M**) Relative RSU-1, GDF15, PINCH1 and MMP13 protein expression respectively after treatment with NSC or RSU-1 siRNA for 48 h in H4 and A172 cells. Quantification was performed using the NIH ImageJ software and the mean band intensity was calculated from two different immunoblots. (**C**,**F**,**I**,**N**) Representative pictures from Western blot displaying RSU-1, GDF15, PINCH1 and MMP13 expression at the protein level after *RSU-1* silencing for H4 and A172 cell lines. (**K**) Relative RhoA activity 48 h post RSU-1 silencing for H4 and A172 cell lines. Asterisks indicate a statistically significant difference (*p* < 0.05) compared to NSC data.

In an attempt to elucidate the interplay between GDF15 and RSU-1 in glioma cell invasion and the molecular pathway involved, we first assessed the invasion capacity of H4, SW1088 and A172 cells using a 3-dimensional (3D) spheroid formation assay in collagen (Supplementary Figure S3) following *RSU-1* silencing. Our results show that H4 and SW1088 cells become more invasive after silencing, whereas the invasiveness of A172 decreases. Also, we tested the expression of GDF15, PINCH1, RhoA, and MMP13 in H4, SW1088 and A172 cells following *RSU-1* silencing for 48 h. As shown in Figure 4, RSU-1 was silenced both at the mRNA (Figure 4A) and protein level (Figure 4B,C). Supplementary Figure S4 shows the effect of RSU-1 silencing at the mRNA expression of SW1088 cells.

Interestingly, a dramatic increase in *GDF15* mRNA expression was observed following RSU-1 silencing in A172 glioma cells, which have low endogenous GDF15 levels (Figure 4D) versus non-specific control (NSC) sample, while a smaller increase was observed in H4 cells, which have higher endogenous GDF15 levels. Western blotting analysis verified the mRNA data at the protein level (Figure 4E,F). Also, in the SW1088 cell line that has lower endogenous RSU-1 levels, no effect was observed in GDF15 expression upon further reduction of RSU-1 mRNA (Supplementary Figure S4B).

Next, we tested the effect of *RSU-1* silencing on *PINCH1*, *RhoA*, and *MMP13* expression. *RSU-1* silencing resulted in upregulation of PINCH1 (Figure 4G), RhoA (Figure 4J) and MMP13 (Figure 4L) in H4 cells and SW1088 cells, respectively (Supplementary Figure S4C–E) and downregulation in A172 cells (Figure 4G–L). RhoA activation assay was also performed 48 h post RSU-1 silencing (Figure 4K) and our results followed the same pattern as *RhoA* mRNA expression in H4 and A172 cell lines (Figure 4J).

### 2.4. GDF15 Silencing Leads to Reduced Invasion in More Invasive Cells but Does Not Affect Less Invasive Cells

**Figure 5:**
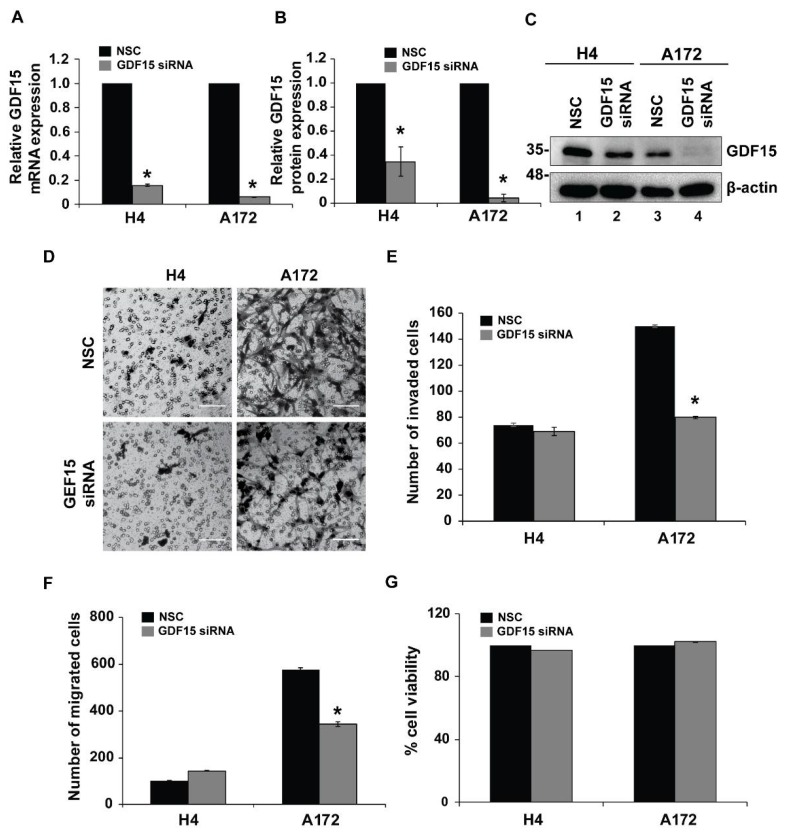
*GDF15* silencing does not interfere with cell survival but inhibits cell invasion and migration of the aggressive cell line (A172), whereas it does not affect the non-invasive cell line (H4). (**A**) Relative *GDF15* mRNA expression in H4 and A172 after NSC or *GDF15* siRNA transfection for 48 h. Three independent real-time PCR experiments were performed and data were analyzed using the ΔΔCt method, with NSC as a calibrator sample for each cell line. (**B**) Quantification of GDF15 protein expression using three different immunoblots. Analysis was performed using NIH ImageJ software. (**C**) Representative picture of Western blot showing the silencing of GDF15 protein after NSC or *GDF15* siRNA for 48 h in H4 and A172. (**D**) NSC and *GDF15* siRNA treated H4 and A172 cells were subjected to invasion assay 24 h post-transfection. Scale bar: 100 μm. (**E**) Diagram showing the total number of invading glioma cells per transwell in each group (nine randomly chosen microscopic fields per transwell). (**F**) Diagram showing the total number of migrated cells per transwell assessed as described above. For invasion and migration assays three independent experiments were performed and each sample was run in duplicate. (**G**) Graph representing the percentage of cell viability assessed by Alamar blue assay 48 h post *GDF-15* siRNA transfection for the two cell lines compared to NSC. Each sample was run in triplicate. Asterisks denote a statistically significant difference (*p* < 0.05) compared to NSC data for each cell line.

**Figure 6:**
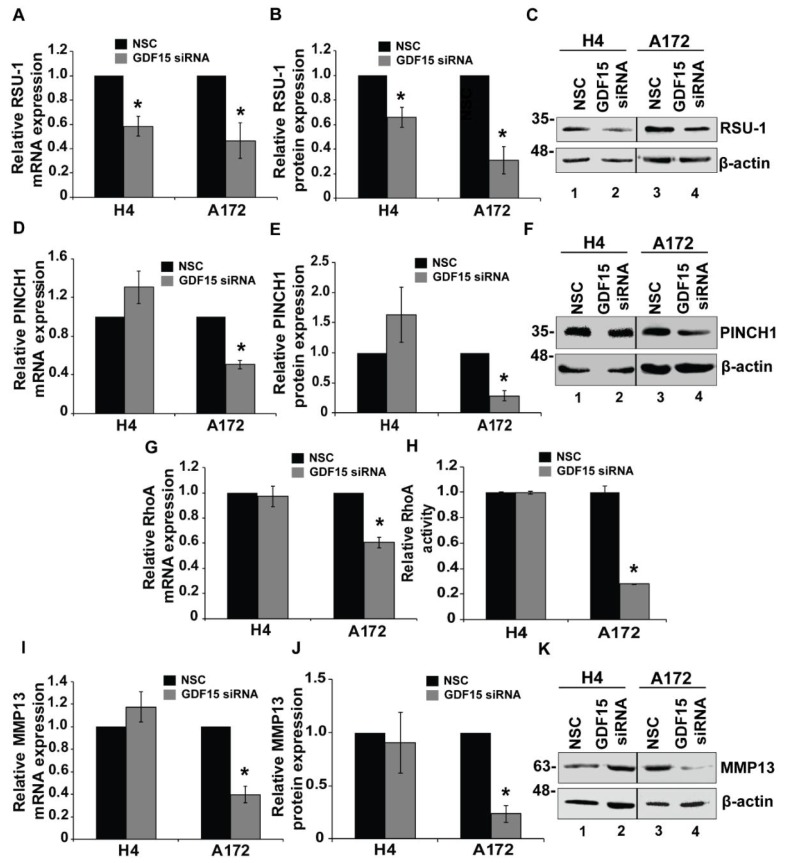
Effect of *GDF15* silencing on *RSU-1*, *PINCH1*, *RhoA* and *MMP-13* expression. (**A**,**D**,**G**,**I**) Relative *GDF15*, *PINCH1*, *RhoA* and *MMP13* mRNA expression respectively in H4 and A172 cells upon *GDF15* silencing. Three independent real-time PCR experiments were conducted and data were analyzed using the ΔΔCt method having control-treated cells as calibrators for each cell line. (**H**) Relative RhoA activity 24 h post rhGDF15 treatment on H4 and A172 cell lines. (**C**,**F**,**K**) Representative image from Western blot analysis displaying RSU, PINCH1 and MMP13 protein expression after *GDF15* silencing. (**B**,**E**,**J**) Graphs representing quantification of RSU-1, PINCH1 and MMP13 protein expression for each cell line using ImageJ software. Two immunoblots from independent experiments were used for the quantification. Asterisks indicate a statistically significant difference (*p* < 0.05) compared to NSC data.

We have shown so far in our study that GDF15-treated cells (H4 and A172) exhibited distinct invasive behavior that was correlated with changes in expression of *RSU-1*, *PINCH1*, *RhoA* and *MMP13*, and that silencing of *RSU-1* leads H4, SW1088 and A172 cells to behave similarly to the GDF15-treated cells with the same alterations in gene expression (*PINCH1*, *RhoA* and *MMP13)*. Subsequently, we investigated the effect of *GDF15* silencing on these parameters. For this purpose, H4 and A172 cell were transfected with NSC or GDF15 siRNA for 48 h. As shown in Figure 5, *GDF15* silencing was successful both at the mRNA (Figure 5A) and protein level (Figure 5B,C). Following *GDF15* silencing, cells were subjected to transwell migration and invasion assays (Figure 5 and Supplementary Figure S5). Our results show that *GDF15* silencing reduced the migration and invasion capacity of the aggressive A172 cells, whereas invasion and migration of H4 cells was not affected (Figure 5D). These results were consistent with 3D spheroids invasion assay (Supplementary Figure S6). Cell viability assay was performed to exclude the possibility of reduced cell migration and invasion due to cell death. As depicted in Figure 5G, cell survival was not affected by *GDF15* silencing further strengthening our findings that *GDF15* silencing inhibits migration and invasion in A172 cells. Moreover, gene expression analysis further corroborated our data showing that although *RSU-1* was downregulated (Figure 6A–C) in both cell lines after *GDF15* silencing, the expression of *PINCH1* (Figure 6D–F), *RhoA* (Figure 6G,H) and *MMP13* (Figure 6I–K) followed a pattern similar to that of cell invasion, being reduced only in A172 cells.

Finally, to better understand the molecular mechanism governing RSU-1 and GDF-15 in glioma cells, we proceeded to silence *RSU-1* for 24 h and then treated the cells with hrGDF-15 for another 24 h. As shown in supplementary Figure S7, hrGDF15 treatment enhances the effects of *RSU-1* siRNA on H4 and A172 cells, suggesting that they have the same end result and they are likely involved in a common signaling pathway.

## 3. Discussion

RSU-1, which binds to PINCH1 at FA sites [19] has been previously found to be upregulated in more invasive breast and brain cancer cells [29,34]. Moreover, we recently demonstrated a differential regulation of cell migration and invasion of glioma cells by RSU-1 based on their aggressiveness, with RSU-1 promoting an invasive behavior in aggressive cells (A172 and U87-MG) and inhibiting them in the less aggressive ones (H4 and SW1088) [34]. This by itself indicates the existence of a complex molecular mechanism that governs glioma cell invasion in vitro. Moreover, *GDF15* is downregulated in aggressive glioma cell lines (SW1088 and A172) in contrast to non-aggressive neuroglioma cells (H4) and its expression is exactly opposite from that of *RSU-1* in glioma cells (Figure 1). This, combined with the fact that *GDF15* is involved in actin cytoskeleton organization [41], prompted us to investigate the interplay between this protein and RSU-1 with regard to glioma cell aggressiveness in vitro. Although, there are many studies documenting the role of *GDF-15* on proliferation, invasion and migration of cancer cells [47,48,50], these results are contradictory tending to indicate that its function is, to a certain extent, cell type-specific. Here, we provided evidence for the role of *GDF15*, in glioma cell invasion and for its correlation with RSU-1 in this process.

**Figure 7:**
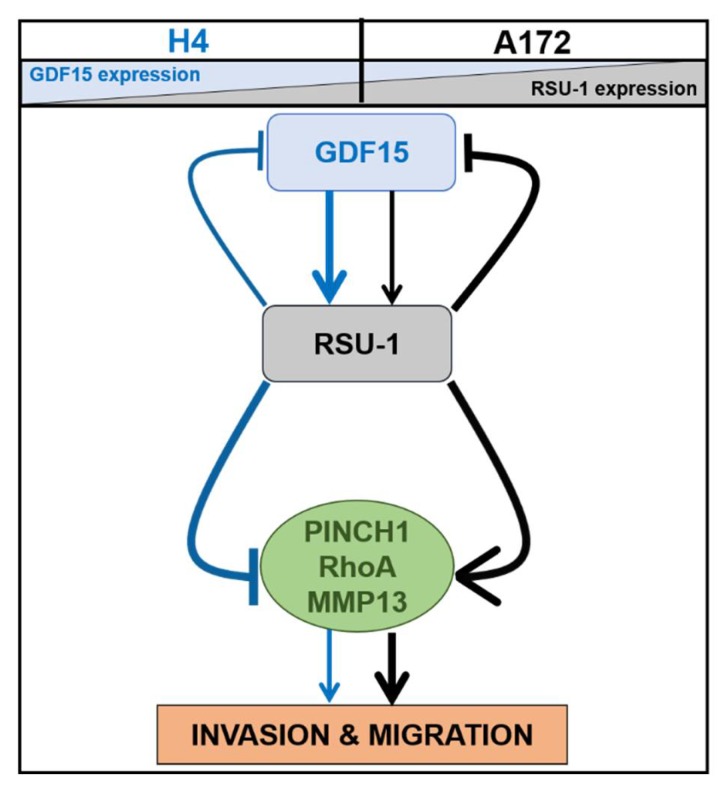
Schematic representation of the involvement of *RSU-1* and *GDF15* in regulating of H4 and A172 cell migration and invasion. The non-invasive H4 cells, endogenously express high levels of GDF15 and low levels of RSU-1 in contrast to the highly invasive A172 cells which endogenously express high RSU-1 and low GDF15 levels. This differential expression leads to a different behavior with regard to brain cell migration and invasion. In H4 cells, *GDF15* induces *RSU-1*, which in turn inhibits migration and invasion by inhibiting *PINCH1*, *RhoA* and *MMP-13* (solid blue arrows and lines). In the invasive A172 cells, *GDF15* promotes *RSU-1* which enhances migration and invasion through upregulation of *PINCH1*, *RhoA*, and *MMP-13* (solid black arrows and lines). Arrows used in the diagram are of different weight, so that thick arrows indicate stronger effect while thin arrows indicate weaker effect, further emphasizing the concept that expression levels of both RSU-1 and GDF15 are crucial in regulating glioma cell migration and invasion.

To investigate the interplay and possible connection between *RSU-1* and *GDF15* in glioma cells, we used three different brain cell lines, namely H4, SW1088 and A172, which have different tumoral origin, properties and proteins expression level [34,56,57]. and four different experimental approaches; (a) hrGDF15 treatment, (b) *RSU-1* silencing, (c) *GDF15* silencing, and (d) combined hrGDF15 treatment and *RSU-1* silencing. In all four approaches, the expression of *RSU-1*, *GDF15*, *PINCH1*, *RhoA* and *MMP-13* as well as the effect on cell migration and invasion was investigated. Figure 7 presents a diagrammatic summary of the molecular interactions, based on our findings. As shown in the diagram, in cells with high GDF15 and low RSU-1 expression (H4 cells), hrGDF15 treatment upregulates *RSU-1* (which is very low at an endogenous level), *PINCH1*, *RhoA* and *MMP-13* and promotes migration and invasion, whereas in cells with low GDF15 and high RSU-1 expression (A172 cells) the effect is the opposite. Interestingly, in SW1088 cells with intermediate expression level of RSU-1 and GDF15, insignificant changes were noticed following hrGDF15 treatment both with regard to invasion (Figure 2B) and migration (Figure 2C) as well as gene expression (Supplementary Figure S2). Thus, *GDF15* promotes invasion in H4 cells and inhibits it in A172 through alterations in *PINCH1*, *RhoA* and *MMP-13* expression, which are known to regulate cell migration and invasion.

To test the hypothesis that *GDF15* and *RSU-1* are implicated in the same molecular mechanism to regulate H4, SW1088 and A172 cells invasion, we silenced *RSU-1* (Figure 4) and observed a strong upregulation of *GDF15* in the A172 cell line, which has low endogenous GDF15 levels (Figure 4D–F) and a smaller change was observed in H4 cells in which GDF15 is abundant. These results suggest that RSU-1 inhibits GDF15 in A172 cells in which *RSU-1* expression levels are higher and GDF15 treatment promotes *RSU-1* expression in H4 cells in which GDF15 levels are higher. Also, the SW1088 cell line has intermediate motility behavior and this is in accordance with the relative endogenous level of RSU-1 and GDF15.

By silencing *RSU-1*, we showed that migration and invasion (Supplementary Figure S3) are increased in H4 and decreased in A172 [34] suggesting that *RSU-1* by itself inhibits migration and invasion in H4 and promotes them in A172 again through regulation of *PINCH1*, *RhoA* and *MMP-13* expression (Figure 4). This is not surprising as *PINCH1* is in direct interaction with *RSU-1* and of course it is very possible any change in *RSU-1* expression also affects *PINCH1* [19]. Moreover, RhoA is known to promote the formation of actin fibers and to be implicated in cell migration [58] and as it is shown in Figure 3H, its activation was following the same pattern with the mRNA expression after hrGDF15 treatment.

In an effort to investigate the exact molecular mechanism underlying the *RSU-1* function by *GDF15* regulation, we finally silenced *GDF15* by siRNA-mediated silencing (Figure 5). Our results show that *GDF15* silencing inhibited cell migration and invasion of A172 cells and did not affect the mobility of H4 cells. As shown in Figure 7, if GDF15 is missing from the pathway then migration and invasion are regulated by RSU-1, which differentially regulates them in the two cell lines. Interestingly, the expression of *PINCH1*, *RhoA* and *MMP13* also followed identical pattern (Figure 6D–G,I–K), while *RSU-1* was downregulated upon *GDF15* silencing in both cell lines (Figure 6A–C). These results suggest that *GDF15* silencing in the less invasive H4 cells with lower RSU-1 expression downregulates *RSU-1* further without affecting invasion and gene expression. On the other hand, *GDF15* silencing in the more invasive A172 cells leads to reduced *RSU-1* expression that is in agreement with the cell invasion pattern being also consistent with the findings obtained from direct *RSU-1* silencing (Figure 4).

To summarize, the data presented in this study provide evidence that there is a strong connection between *RSU-1* and *GDF15* in H4 and A172 cells, which is different from the one observed in breast cancer cells [54], further corroborating the idea that GDF15’s function is, to a great extent, cell-type specific. More importantly, this work points out the significance of the relative expression of these two proteins in affecting the ability of cells to migrate and invade in brain parenchyma. Moreover, our knowledge of the molecular mechanism in which GDF15 and RSU-1 are involved will facilitate the identification of therapeutic targets in signaling pathways that are crucial to cancer development and progression. Future studies are thus needed in order to better clarify the exact mechanism in which *RSU-1* and *GDF15* take part in gliomas and evaluate the diagnostic potential of their expression levels.

## 4. Materials and Methods 

### 4.1. Glioma Cell Lines

H4, SW1088 and A172 human cells were obtained from American Type Culture Collection (ATCC). H4 is a neuroglioma cell line with an epithelial morphology, SW1088 is an astrocytoma cell line with a fibroblast like morphology, while A172 are cells isolated from GBM patients. All cells were grown in high-glucose Dulbecco’s modified Eagle’s medium (DMEM) supplemented with 10% fetal bovine serum and 1% antibiotic/antimycotic and were cultured at 37 °C in a 5% CO_2_ humidified atmosphere.

### 4.2. Antibodies and Reagents

Anti-PINCH1 antibody was purchased from Cell Signaling Technology, anti-β-actin antibody was purchased from Sigma-Aldrich, anti-GDF15 was from Santa Cruz Biotechnology and the anti-RSU-1 rabbit antibody was kindly provided by Dr. Mary Lou Cutler, Professor at the Uniformed Services University of the Health Sciences, Bethesda USA. Anti-MMP13 was purchased from Abcam. *RSU-1* siRNA and GDF-15 siRNA were purchased from Santa Cruz Biotechnology. Lipofectamine 2000 was purchased from Invitrogen Life Technologies and Alamar Blue reagent was obtained from Thermo Scientific. Transwell inserts were obtained from Greiner Bio-One, and Matrigel was from Corning. QIAzol Lysis Reagent was purchased from QIAGEN, GDF15 human recombinant protein (hrGDF15) was obtained from R&D systems, and G-LISA RhoA Activation Assay was purchased from Cytoskeleton. Collagen I was obtained from Corning.

### 4.3. Transwell Migration and Invasion Assays

The same transwell chambers from Corning with membranes of 8μm pore size were used both for migration and invasion assays. In the case of migration assays, the chambers were used uncoated while in the case of invasion assays they were coated with 520 μg/mL Matrigel [59,60]. In total, 3 × 10^4^–3.5 × 10^4^ cells were resuspended in 0.5 mL of serum-free medium and placed in the upper chamber of the transwell. Complete DMEM containing 10% fetal bovine serum and 1% antibiotic/antimycotic was placed in the lower chamber (750 μL)- in 24-well plates. Cells were left to migrate/invade for 24 h at 37 °C and non-migrating/invading cells were removed from the upper surface of each chamber with a cotton swab while the cells that actually migrated or invaded to the lower surface were fixed with 4% PFA for 15 min, and stained with 0.1% crystal violet (dissolved in water) for 30 min [61,62,63]. All inserts were washed three times with ddH_2_O and pictures were taken from nine (9) randomly selected microscopic fields that covered the entire surface of the insert membrane using a Nikon Eclipse optical microscope equipped with a digital camera. Cells that migrated/invaded through the membrane were counted and the sum was taken for all nine optical fields. Experiments were run in duplicate and four independent experiments were performed.

### 4.4. Tumor Spheroids Formation in Collagen Gels

Generation of tumor spheroids was performed using the «hanging drop» method as described previously [29]. Following transfection with NSC or *RSU-1* siRNA, a suspension of 2.5 × 10^4^ was used for generating drops of 20 μL containing 500 cells each [64]. Drops were left for 24 h so that spheroids are formed and spheroids were then embedded in 1 mg/mL collagen I gels inside wells of a 96-well plate [34]. Pictures were taken at time zero and at 6 or 16 h later, depending on the cells’ aggressiveness. The spheroids’ size was determined using the ImageJ software and differences between the time zero and final time point were measured. 

### 4.5. Transfection with siRNA

Cells seeded at a density of approximately 50% were transfected with 100 nM siRNA against *RSU-1*, or *GDF15* or with a control NSC siRNA, using the Lipofectamine 2000 reagent (7 μL per 35 mm dish) according to the manufacturer’s guidelines. Cells were harvested 48 h post-transfection (as this was the time point with the most effective silencing, as shown in Supplementary Figure S8) or replated into transwells 24 h later and left to migrate/invade for an additional 24 h (total 48 h following siRNA transfection) [54].

### 4.6. Treatment with hrGDF15

For the experiments that included treatment with hrGDF15, H4, SW1088 and A172 cells were grown until they reached 70% confluency and were then cultured for 24 h in low serum DMEM supplemented with 0.5% fetal bovine serum (FBS). Cells were then subjected to treatment with hrGDF15 (10 ng/mL) or the control solvent in which hrGDF15 was dissolved (4 mM HCI containing 0.1% bovine serum albumin) for 24 h, as we described previously [54].

### 4.7. Cell Viability Assay

H4 and A172 cells transfected with *RSU-1* or *GDF15* siRNA and H4, SW1088 and A172 cells treated with hrGDF15 were subjected to cell viability assay using the Alamar Blue reagent for at least 2 h, according to the manufacturer’s instructions. The absorbance was then measured using Rayto spectrophotometer at 570 and 600 nm. Finally, results were analyzed and compared to the control samples.

### 4.8. RNA Isolation and Real-Time Polymerase Chain Reaction (PCR)

Total RNA was extracted from cells using QIAzol Lysis Reagent and transcribed to cDNA using Superscript Reverse Transcriptase. Quantification of gene expression was performed by Real Time PCR in a CFX96 Real Time-PCR machine (BioRad) using the ΔΔCt method. The nucleotide sequences of the specific primers used are described in Table 1.

### 4.9. Protein Extraction and Western Blotting

Whole cell extracts were prepared using radio immunoprecipitation assay (RIPA) buffer containing 1% sodium dodecyl and western blot analysis was performed using standard immunoblotting protocols as described previously [29]. More specifically, equal amounts of total protein lysates were separated by 12% polyacrylamide gel electrophoresis. The proteins were transferred to a PVDF membrane using the Semi-dry transfer system (BioRad). Then, the membrane was blocked with 5% skim milk in tris-buffered saline-tween (TBS-T) buffer for 1 h and was incubated with appropriate antibodies overnight in 5% skim milk at 4 °C. The detection of the antibody was performed with enhanced chemiluminescent system from Pierce and Kodak Biomax light films or with ChemiDoc XRS + Imaging System (BioRad) and protein expression was quantified compared to the β-actin loading control using the National Institute of Health (NIH) ImageJ software. The mean intensity of respective protein bands from three different immunoblots was used for the quantification, as indicated.

### 4.10. RhoA Activation Assay

RhoA activation was assessed using the G-LISA RhoA activation assay kit (Cytoskeleton) according to the manufacturer’s instructions.

### 4.11. Statistical Analysis

All results were represented as mean ± standard error (SE). Significant changes were determined by Student’s *t*-test using two-tail distribution or by paired one-way analysis of variance (ANOVA) using the software program GraphPadPrism (6.0 for Windows; GraphPad Prism Software Inc., San Diego, CA, USA). Differences with *p* values < 0.05 were considered as statistically significant (indicated by an asterisk *).

## 5. Conclusions

GBM is the most aggressive type of brain tumor [3,65]. RSU-1 localizes to cell-ECM adhesion sites through its interaction with PINCH-1 [66] and has been shown to promote the metastatic behavior in breast and liver cancer cells in vitro [28,29]. Interestingly, we recently found that it differentially regulates glioma cell invasion based on the cells’ aggressiveness [34]. GDF15, a member of the TGF-β family of proteins, known to be involved in actin cytoskeleton reorganization, is elevated in glioblastoma patients’ serum [41,48]. In the present study, we focused on the interplay between RSU-1 and GDF15 and their role in regulating glioma cell invasion using three different glioma cell lines (H4, SW1088 and A172). These cell lines were of increasing invasion capacity while at the same time had opposite patterns of RSU-1 and GDF15 expression levels. We showed that glioma cells behave differently with regard to cell migration and invasion depending on the relative *RSU-1* and *GDF15* expression. Also, we found that PINCH1, RhoA and MMP13 play a crucial role being regulated by the RSU-1/GDF15 interplay leading to affection of the final invasive phenotype of glioma cells. This is the first work showing a strong connection between RSU-1 and GDF15 in malignant H4 and A172 cells and provides the basis for their future evaluation as novel anti-invasive targets in gliomas.

## Figures and Tables

**Table 1:** Primer sequences used for real time PCR.

Primer Name	Sequence
GDF15	Forward: 5′- TCAAGGTCGTGGGACGTGACA-3′ Reverse: 5′-GCCGTGCGGACGAAGATTCT-3′
MMP13	Forward: 5′-TGGCATTGCTGACATCATGA-3′ Reverse: 5′-GCCAGAGGGCCCATCAA-3′
PINCH1	Forward: 5′-CCGCTGAGAAGATCGTGAAC-3′ Reverse: 5′-GGGCAAAGAGCATCTGAAAG-3′
RhoA	Forward: 5′-CGGGAGCTAGCCAAGATGAAG-3′ Reverse: 5′-CCTTGCAGAGCAGCTCTCGTA-3′
RSU-1	Forward: 5′-AGGCCACAGAGCAAGGTCTA-3′ Reverse: 5′-CGTGCAATCTCAAAAGCTCA-3′
β-actin	Forward:5′-CGAGCACAGAGCCTCGCCTTTGCC-3′ Reverse: 5′-TGTCGACGACGAGCGCGGCGATAT-3′

## Supplementary Materials

The following are available online at https://www.mdpi.com/2072-6694/11/8/1159/s1: Figure S1: Cell migration in H4, SW1088 and A172 cells, Figure S2: Relative mRNA expression, Figure S3: Tumor spheroid invasion assay after NSC and RSU-1 siRNA transfection in spheroids embedded in collagen I gels, Figure S4: Relative mRNA expression in SW1088 cells following RSU-1 silencing, Figure S5: Cell migration in H4 and A172 cells following GDF15 silencing, Figure S6: Tumor spheroid invasion assay after NSC and GDF15 siRNA tansfection in spheroids embedded in collagen I gels, Figure S7: Combination of RSU-1 silencing and treatment with hrGDF15 (10 ng/mL) has the same effect with RSU-1 silencing on its own on transwell invasion assay and on RSU-1 expression for H4 and A172 glioma cell lines, Figure S8: RSU-1 and GDF15 silencing.
