# Supplementary material for: Coordinated Expression of Ras Suppressor 1 (RSU-1) and Growth Differentiation Factor 15 (GDF15) Affects Glioma Cell Invasion
Source: Cancers (Basel). 2019 Aug 13;11(8):1159. doi: 10.3390/cancers11081159 (PMC6721804; doi:10.3390/cancers11081159)
Supplement: Supplementary file 1 [file cancers-11-01159-s001.zip › cancers-568542-SI.pdf]

Supplementary Materials: Coordinated Expression of Ras Suppressor 1 (RSU-1) and Growth Differentiation Factor 15 (GDF15) Affects Glioma Cell Invasion

Maria Louca, Vasiliki Gkretsi and Triantafyllos Stylianopoulos

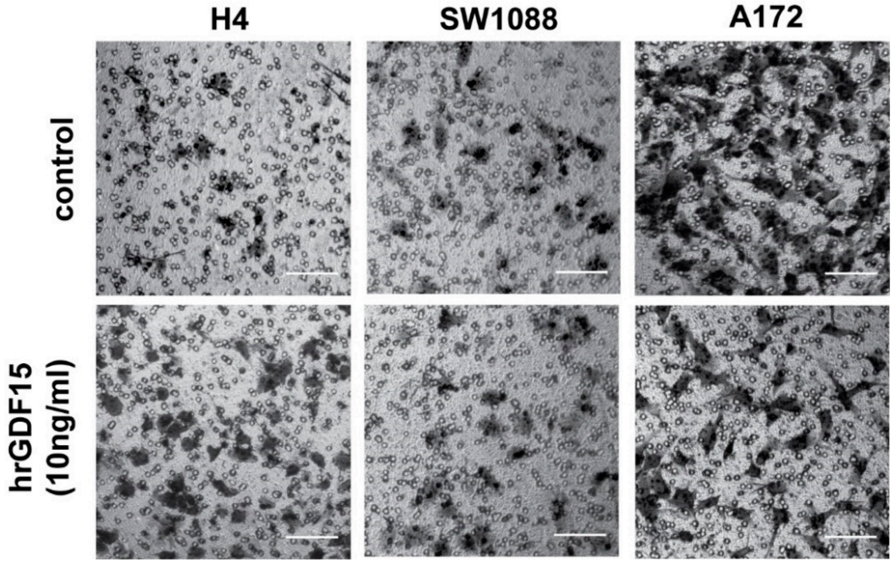

Figure S1. Cell migration in H4, SW1088 and A172 cells. Representative images (control and hrGDF15-treated cells (10 ng/mL)), taken with Nikon Eclipse TS100 optical microscope. Three (3) independent experiments were done and each sample run in duplicate. Scale bar: 100 μm.

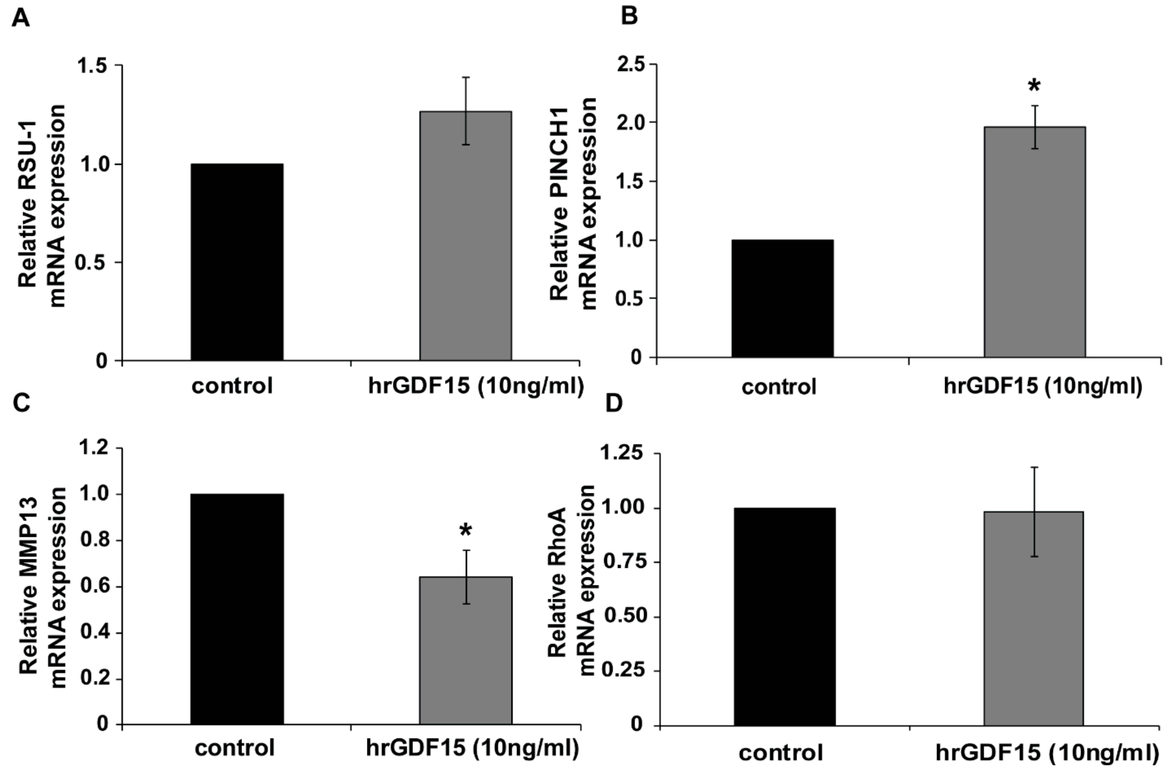

Figure S2. Relative mRNA expression following hrGDF15 treatment for *Rsu-1* **(A)**, *PINCH1* **(B)**, *MMP13* **(C)** and *RhoA* **(D)** for SW1088 cell line 48 h post -hrGDF15 treatment. Two (2) independent experiment were performed and quantification was done using a control as the calibrator sample. Asterisks denote statistically significant changes ($p < 0.05$) compared to control data.

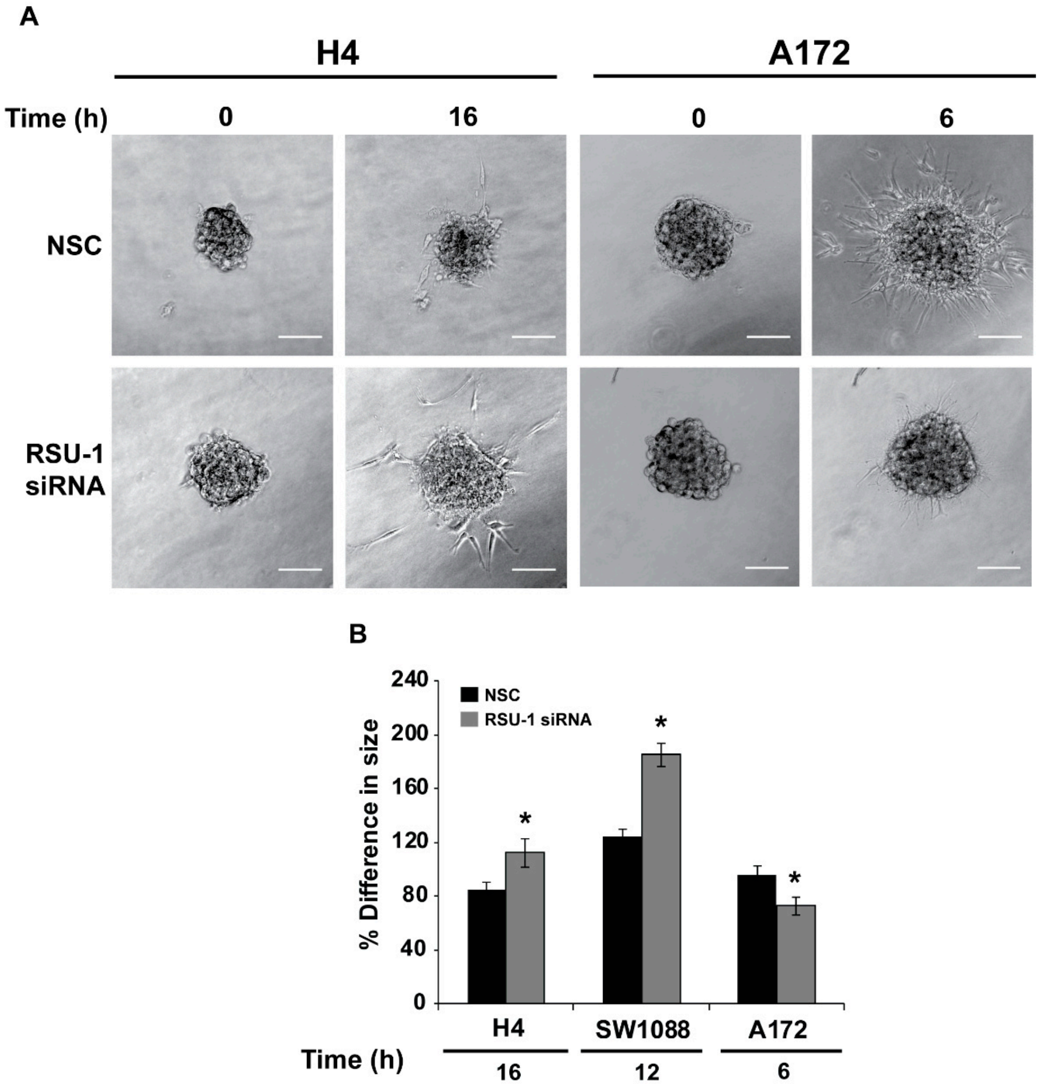

Figure S3. Tumor spheroid invasion assay after NSC and *RSU-1* siRNA transfection in spheroids embedded in collagen I gels. Spheroids (in average $n = 20$ spheroids per cell line) were embedded in 1 mg/mL collagen I gel and left to invade through the gel for different time periods depending on the aggressiveness of each cell line. **(A)** Representative images of H4 (least invasive) and A172 (most invasive) cells for 16 h and 6 h, respectively. Scale bar: 100 μm. **(B)** The percentage of tumor spheroid invasion for H4, SW1088 and A172 cell line was assessed by measuring the difference of each spheroid size ((major + minor axis)/2) within the corresponding hours following placement of the spheroid in the collagen gel (time zero). Three (3) independent experiment were performed and quantification was done using the NSC as the control sample. Asterisks denote statistically significant changes ($p < 0.05$) compared to NSC data.

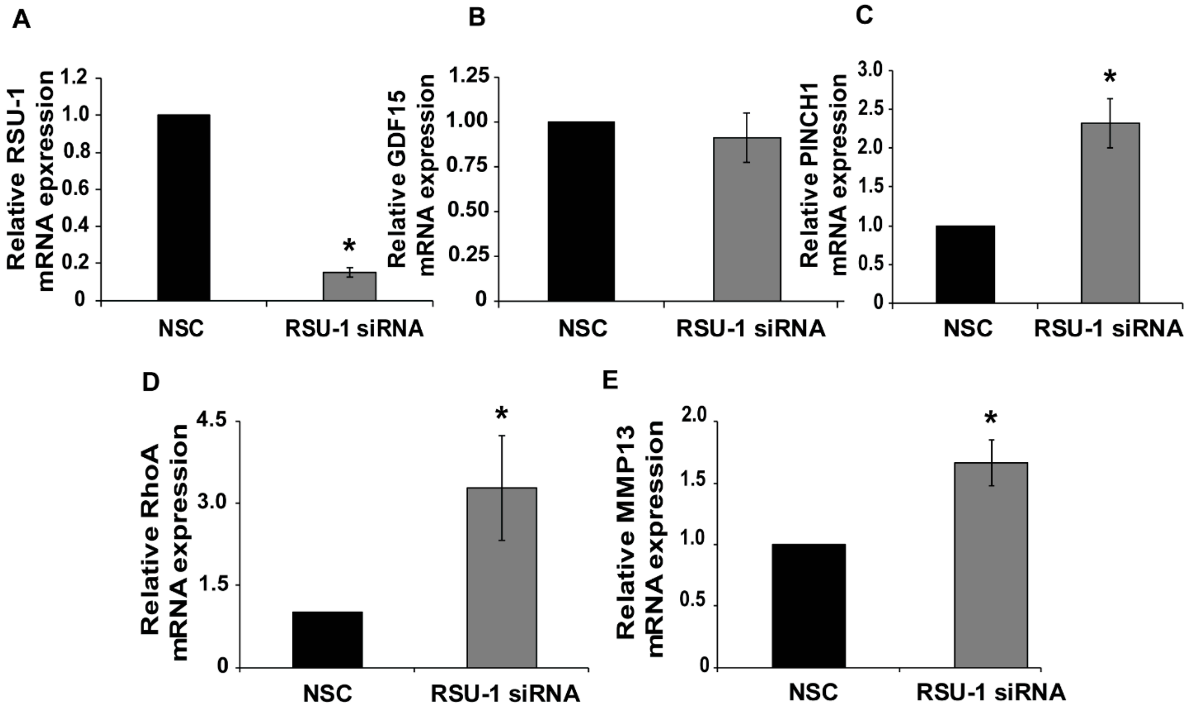

Figure S4. Relative mRNA expression in SW1088 cells following *RSU-1* silencing for *RSU-1* **(A)**, *GDF15* **(B)**, *PINCH1* **(C)**, *RhoA* **(D)** and *MMP13* **(E)** 48h post treatment with NSC and *RSU-1* siRNA. At least Four (4) independent experiment were performed and quantification was done using the NSC as the calibrator sample. Asterisks symbolize statistically significant changes ($p < 0.05$) compared to control data.

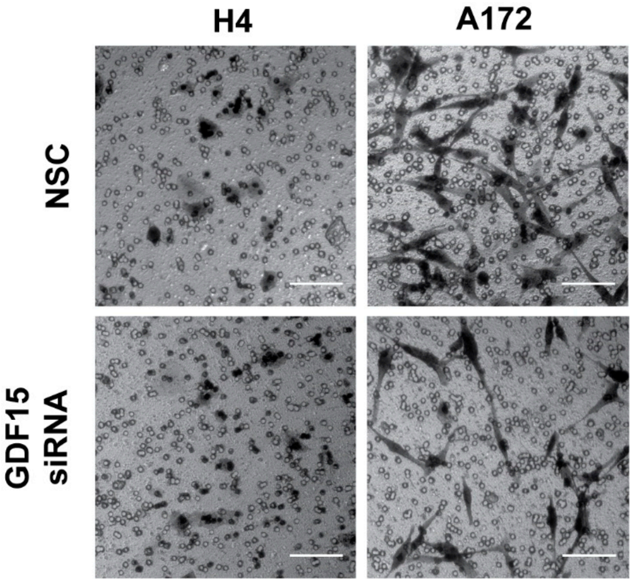

Figure S5. Cell migration in H4 and A172 cells following *GDF15* silencing Representative images (NSC and upon *GDF15* siRNA transfection), taken using Nikon Eclipse TS100 optical microscope. Three (3) independent experiments were performed and two transwells were used per sample. Scale bar: 100 μm.

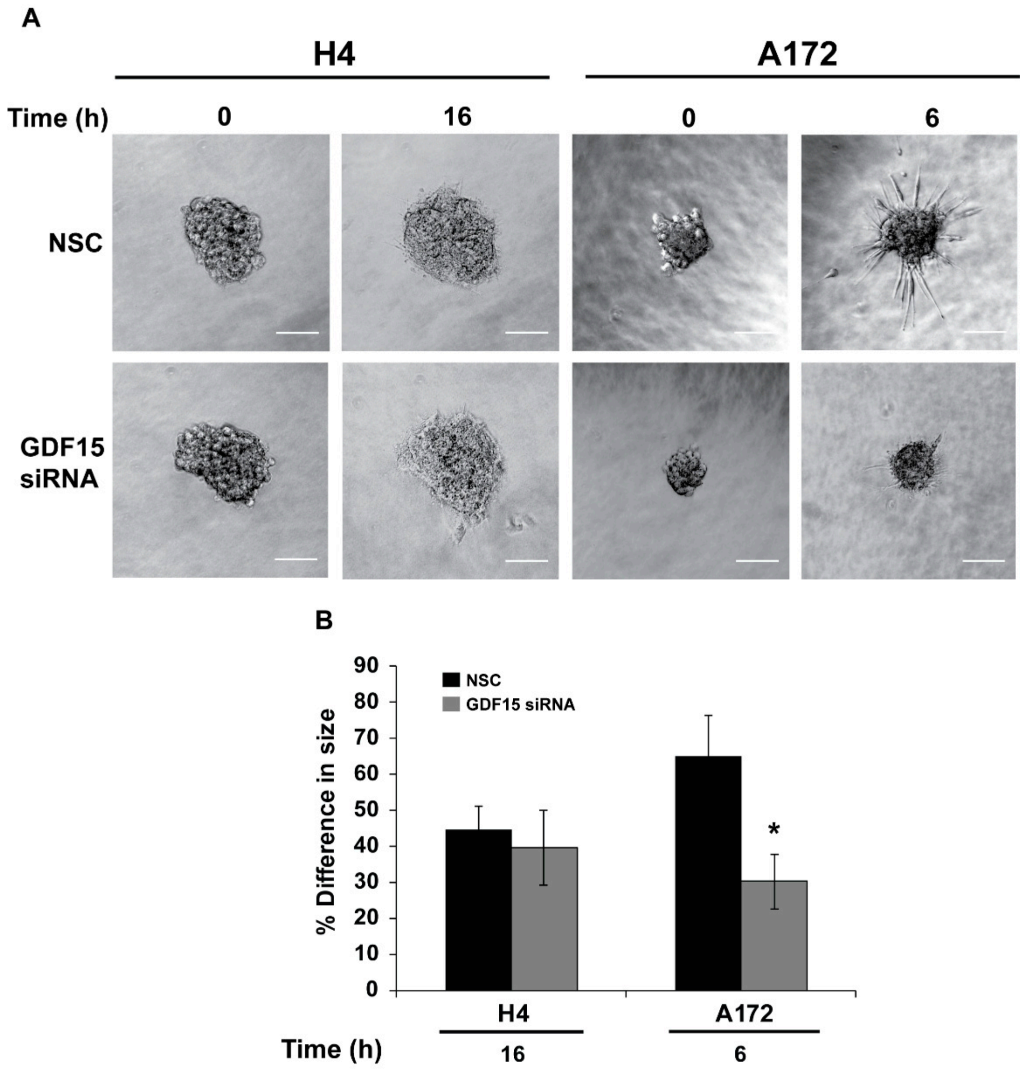

Figure S6. Tumor spheroid invasion assay after NSC and *GDF15* siRNA tansfection in spheroids embedded in collagen I gels. Spheroids (in average n = 10 spheroids per cell line) were embedded in 1mg/ml collagen I gel and left to invade through the gel for different time periods depending on the aggressiveness of each cell line. Scale bar: 100 μm. **(A)** Representative images of H4 (least invasive) and A172 (most invasive) cells for 16h and 6 h, respectively. **(B)** The percentage of tumor spheroid invasion for H4 and A172 cell line was assessed by measuring the difference of each spheroid size ((major + minor axis)/2) within the corresponding hours following placement of the spheroid in the collagen gel (time zero). Two (2) independent experiment were performed and quantification was done using the NSC as the control sample. Asterisks denote statistically significant changes ($p < 0.05$) compared to NSC data.

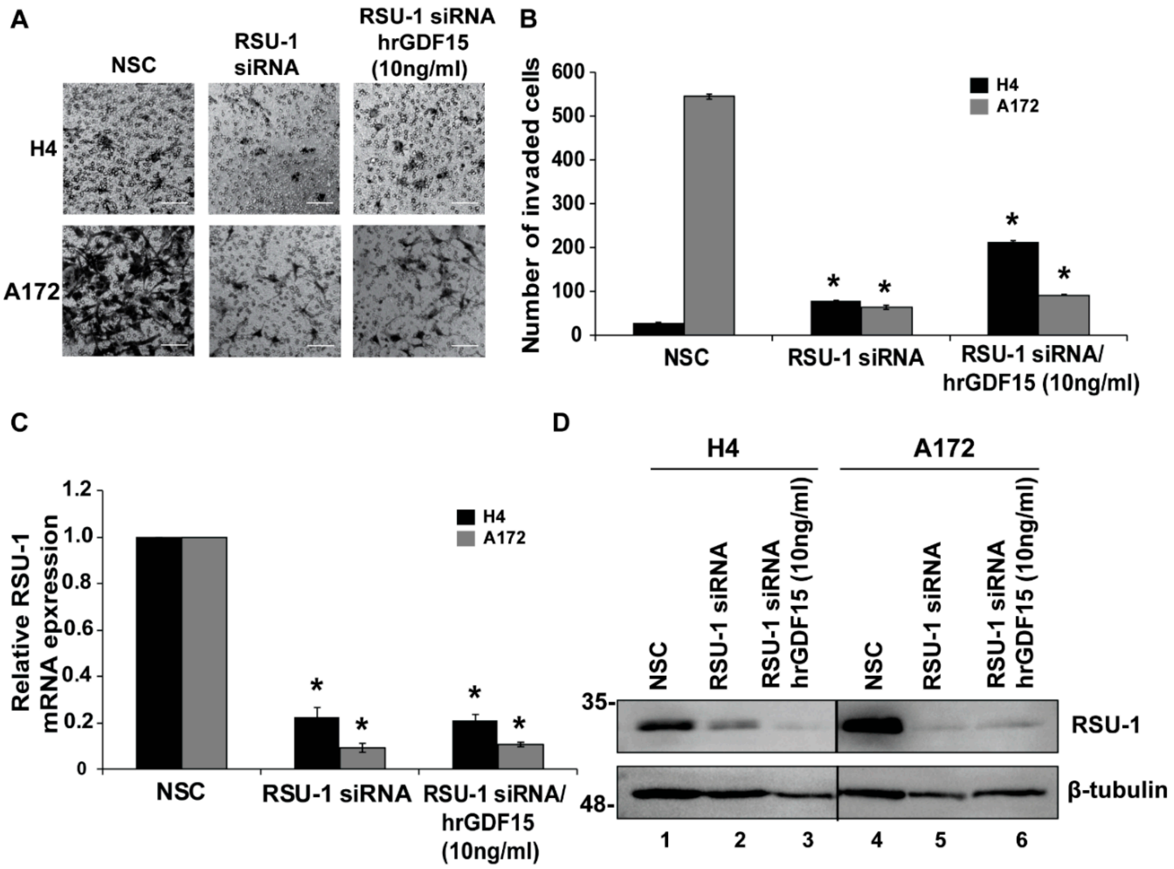

Figure S7. Combination of *RSU-1* silencing and treatment with hrGDF15 (10 ng/mL) has the same effect with *RSU-1* silencing on its own on transwell invasion assay and on RSU-1 expression for H4 and A172 glioma cell lines. **(A)** Representative images of a transwell invasion assay that was performed for 24h for H4 and A172 cell lines with NSC, *RSU-1* siRNA, or the combination *RSU-1*siRNA and treatment with hrGDF15 (10 ng/mL). The invading cells were counted in nine (9) randomly chosen microscopic field per transwell. Scale bar: 100 μm. **(B)** Total number of invaded cells compared to NSC for *RSU-1* siRNA and the *RSU-1* siRNA for *RSU-1* siRNA/GDF15 treatment (10 ng/mL) for each cell line per transwell. Each sample was run in duplicate and two (2) independent experiments were performed. **(C)** Relative *RSU-1* mRNA expression for H4 and A172 cell lines with NSC, *RSU-1* siRNA, or the combination *RSU-1*siRNA and treatment with hrGDF15 (10 ng/mL). Three independent Real-Time PCR experiments were performed and data were analyzed using the ΔΔCt method, while NSC treated cells were used as the calibrator sample for each cell line. **(D)** Representative pictures from Western blot analysis displaying RSU-1 expression at the protein level following transfection with NSC, *RSU-1* siRNA, or the combination *RSU-1*siRNA and treatment with hrGDF15 (10 ng/mL) for H4 and A172 cell lines. Asterisks symbolize statistically significant changes ($p < 0.05$) upon *RSU-1* siRNA and *RSU-1*siRNA/ hrGDF15 compared to NSC sample for each cell line.

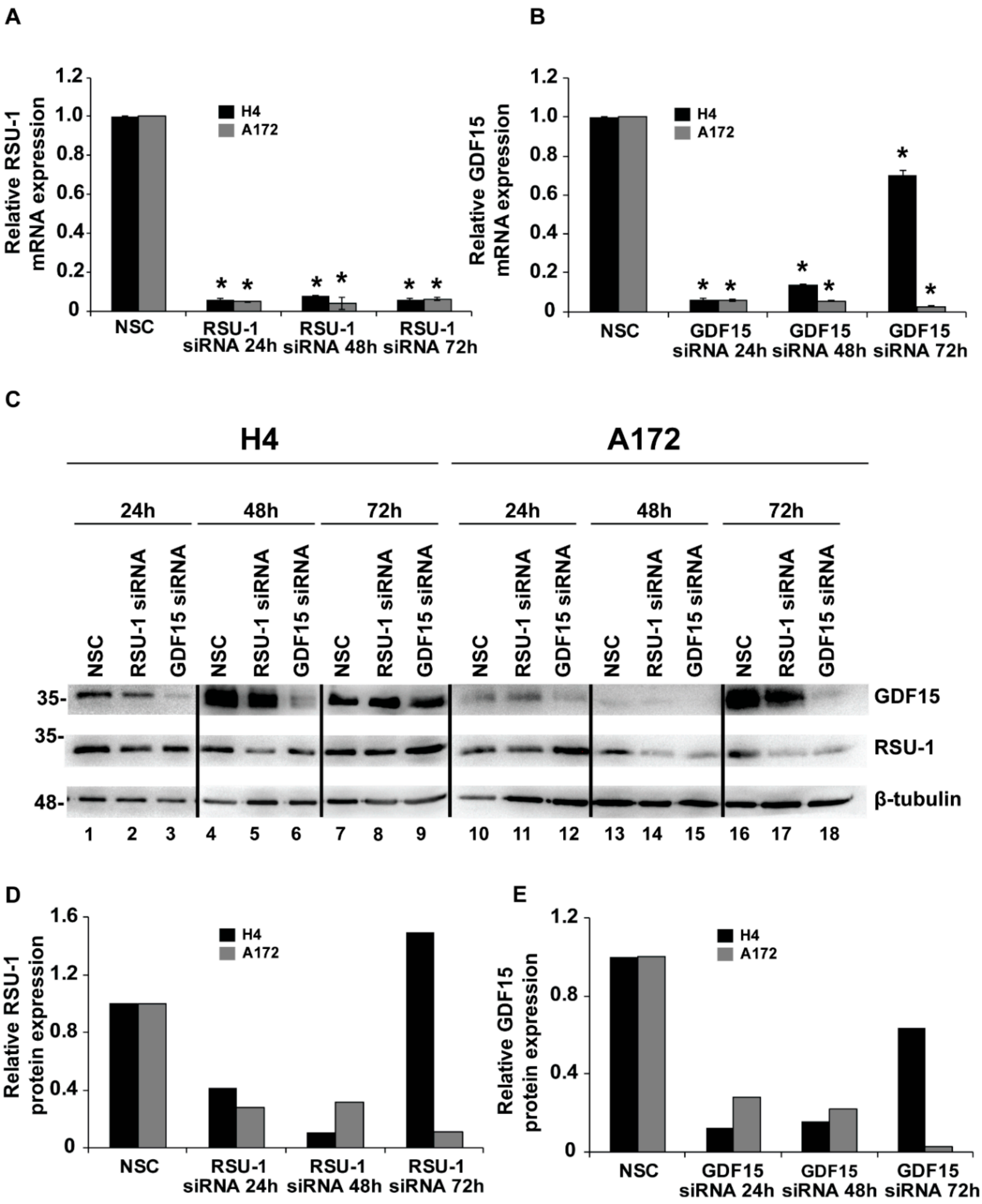

Figure S8. RSU-1 and GDF15 silencing **(A&B)** Relative *RSU-1* and *GDF15* mRNA expression in H4 and A172 cells upon RSU-1 and GDF15 silencing respectively for three different time points (24 h, 48 h and 72 h). Asterisks denote a statistically significant difference ($p < 0.05$) compared to the NSC data for each time point and cell line. **(C)** Representative image from Western blot analysis displaying the efficiency of *RSU-1* and *GDF15* silencing for each time point and cell line used. **(D&E)** Graphs representing quantification of RSU-1 and GDF15 protein expression **(C)** for each time point and cell line using the ImageJ software.

© 2019 by the authors. Licensee MDPI, Basel, Switzerland. This article is an open access article distributed under the terms and conditions of the Creative Commons Attribution (CC BY) license (http://creativecommons.org/licenses/by/4.0/).
